# Accepting telemedicine in a circulatory medicine ward in major hospitals in South Korea: patients’ and health professionals’ perception of real-time electrocardiogram monitoring

**DOI:** 10.1186/s12913-018-3105-y

**Published:** 2018-04-20

**Authors:** Seo-Joon Lee, Tae-Young Jung, Tae-Ro Lee, Jae-Hoon Han

**Affiliations:** 1 Research Institute of Health Science, Korea University, Anam-ro, Seongbuk-gu, Seoul, Korea; 2 Department of Medical & Health Administration, U1 University, 310, Daehak-ro, Yeongdong-eup, Yeongdong-gun, Chungcheongbuk-do, Korea; 3 BK21 PLUS, School of Health Policy and Management, Graduate School, Korea University, Anam-ro, Seongbuk-gu, Seoul, Korea; 4 Strategic Planning Team, Seoul St. Mary’s Hospital, The Catholic University of Korea, 222, Banpo-daero, Seocho-gu, Seoul, Korea; 5 Department of Medical Information, The Catholic University of Seoul, 222, Banpo-daero, Seocho-gu, Seoul, 06591 Korea

**Keywords:** Telemedicine, Tertiary care centers, Perception, Cardiology, Electrocardiography

## Abstract

**Background:**

South Korean government is currently in progress of expanding the coverage of telemedicine projects as part of an attempt to vitalize service industry, but is facing fierce opposition from KMA. Practice of telemedicine requires sufficient discussions among related parties. Although the participation of medical specialists is important, agreement from the public is essential.

**Methods:**

Three main tertiary care centers in Seoul were selected for data collection. A total of 224 patients (patients *n* = 180, patient guardian *n* = 44) and medical professionals (*n* = 41) were selected using simple random sampling. Mixed method of quantitative survey and qualitative semi-interview was used.

**Results:**

This study analyzed patients’ and medical professionals’ perception about the application of telemedicine in cardiology ward in tertiary care centers to provide baseline data when developing and applying telemedicine services. Results implied high need for encouraging telemedicine projects in order to appeal needs among population by providing experience (*p* < 0.001) and knowledge (*p* < 0.001). Other results showed that the need for electrocardiography monitoring was high among not only in remote areas but also in areas close to the capital. 64.52% of all participants thought that telemedicine was needed, and 73.21% of participants were willing to use telemedicine service if provided. Semi-interviews revealed that participants expected more cost and time saving services through remote treatment, by not having to visit long distance hospitals frequently.

**Conclusions:**

Research results oppose Korean Medical Association’s opinion that the population is against enforcing telemedicine related laws. The findings in this study reflect an up-to-date perception of telemedicine among patients and medical professionals in a tertiary care centers’ cardiology ward. Moreover, the study provides a baseline that is needed in order to overcome past failures and to successfully implement telemedicine in South Korea.

**Electronic supplementary material:**

The online version of this article (10.1186/s12913-018-3105-y) contains supplementary material, which is available to authorized users.

## Background

Internationally, the Ministry of Health and Welfare of South Korea signed a memorandum of understanding (MOU) with Mongolia including telemedicine and e-health cooperation in July 2016 [1]. In addition, the Minister of Health and Welfare announced future plans that South Korea’s tertiary care centers will start to export telemedicine to Peru, the Philippines, and China by October 2016 [2].

Currently, the Korean government is in the process of expanding the coverage of telemedicine projects as part of an attempt to vitalize the service industry [3]. It is expected to create new convergence services regarding healthcare and technology, leading to an increase in job creation and an economic boost.

However, the government has been facing fierce disapproval of telemedicine from opposition groups. The Korean Medical Association (KMA) is especially discontent with the government’s attempt to implement telemedicine. This is not the first time, a few years ago, the Ministry of Health and Welfare had to ultimately withdraw their telemedicine policy plans that started in 2013 due to the general strike by KMA which led to a temporary shutdown of most of the hospitals in Korea. Although not a political party, the association has great influence in the field of medicine.

The KMA’s opinion is that telemedicine is part of a conspiracy to implement healthcare privatization [4], which will drastically increase healthcare costs for domestic citizens. Another claim is that there is not enough agreement between the public, medical professionals, and government about the structure of the medical industry or health policy regarding telemedicine [5]. They also argue that telemedicine is not needed considering that Korea has high accessibility of hospitals.

The problem is that successive conflicts are hindering South Korea from adopting telemedicine in all medical fields. South Korea has been conducting telemedicine-related pilot projects ever since the 1990s, which is a global head start. However, Korea is now lagging behind by more than 20 years compared to Japan regarding telemedicine regulations [6].

Global [7] and domestic research studies [8] and solutions are being developed in medical industry sectors to support telemedicine. Especially in the field of cardiology, the electrocardiography (ECG) has been the core subject of remote health monitoring by renowned corporations such as Scanadu [9], Google [10], and Apple [11] due to its comparatively simple, non-invasive sensors.

The practice of telemedicine requires sufficient discussion among the related parties and, although the participation of medical specialists is important, agreement from the public is essential. The purpose of this study was to analyze the perceptions of patients and medical professionals about the application of telemedicine in the cardiology field to provide useful insights when developing and applying diverse telemedicine services. The research results are envisioned to be used as baseline data for the effective implementation of telemedicine in South Korea under the current circumstances of social conflict.

## Methods

### Design

Domestic research by Jung et al. [12], Cho et al. [13], and Han et al. [14] was used for reference when constructing the survey questionnaire. Foreign research by Bradford et al. [15] was also used for insight. The proposed research was conducted following the Consolidated Criteria for Reporting Qualitative Research [16]. The researchers in charge of this study were academics with at least 5 or more years of professional experience in healthcare.

### Sample and data collection

Cardiology ward tertiary care centers in Seoul, G Hospital, K hospital, and S hospital were selected for data collection. These were top tertiary care centers of Korea (approximately 1300 beds, 1100 beds, and 1356 beds, respectively), and were selected because these evenly cover the medical service range of the capital geographically. The survey began in February of 2016 and ended in April 2016. Korea has a healthcare structure in which fewer than 10 large tertiary care centers cover most patients in the country, therefore selecting major tertiary care centers supports representativeness in this study. A total of 224 patients and 41 medical professionals were selected using simple random sampling [17] to avoid bias (medical professionals looked after patients in a ratio of approximately 1:5). Individuals aged 18 years or younger were excluded because they were considered too young to understand the research and the survey.

Only those people who gave their consent were obliged to answer. A mixed method of both quantitative and qualitative approaches was used. In specific, open-ended questions were included within the questionnaire to account for some in-depth views.

None of the researchers were in any sort of relationship with the participants throughout the study. Ethical approval was given by the Institutional Review Board (IRB), number KC15EISI0103.

### Variables

Note that the original questionnaire was in Korean and the following questions were translated for convenience. Refer to [Additional file 1] for English translation extracted from the original survey. Open-ended questions that were included within the questionnaire to obtain in-depth qualitative opinions of participants were as follows: (1.3A) reason for thinking that conventional ECG testing is not needed (“Please write a short statement about why you think that conventional ECG testing is not needed”), (1.3B) reason for thinking that conventional ECG testing is needed (“Please write a short statement about why you think that conventional ECG testing is needed”), (part of 2.2) other functions of remote monitoring that the respondent thinks are important, (2.3A) reason for agreeing to use remote ECG monitoring (“Please write a short statement about why you will use remote ECG monitoring”), (2.3B) reason for disagreeing to use remote ECG monitoring (“Please write a short statement about why you will not use remote ECG monitoring”), and (part of 2.6) other causes that hinder remote ECG monitoring.

### Statistical analyses

The Statistical Package for the Social Sciences (SPSS) software version 21.0 (IBM Corp., Armonk, NY, USA) was used for all statistical analyses. The descriptive analysis (for results of socio-demographic characteristics and perceptions), correlation analysis (for assessing the relationships between independent and dependent variables prior to regression analyses), single linear regression analysis (between independent variables usage experience and degree of knowledge, and dependent variable perceived need), and multiple regression analysis were used to examine the participants’ perceptions. All infinite decimals were rounded off to two decimal places, except for *p*-values, which were rounded off to three decimal places.

## Results

### Socio-demographic characteristics

A total of 265 participants were included in the statistical analyses. Table 1 shows the general socio-demographic characteristics of all included participants. Most participants were 60 years or older (26.79%). Although most of the participants were from Seoul (57.74%) and Gyeonggi (outer cluster part of Seoul, 16.98%), a considerable number was from other more distant parts of the country (Gangwon, Chungcheong, Jeonla, and Gyeongsang 25.28%). Regarding the type of participant, most were patients and patient guardians (67.92% and 16.60% respectively).

**Table 1:** Socio-demographic characteristics of the participants

Factor	Category	Weighted (%)	*N*
Age (years)	19 to 29	11.70	31
	30 to 39	21.13	56
	40 to 49	23.02	61
	50 to 59	17.36	46
	60 or older	26.79	71
Academic level	Middle school	10.94	29
	High school	14.34	38
	Undergraduate	51.32	136
	Graduate or higher	23.40	62
Income^a^	< 1 mil	10.70	31
	1 mil ≤ < 2 mil	12.83	34
	2 mil ≤ < 3 mil	32.45	86
	3 mil ≤ < 4 mil	17.36	46
	4 mil ≤ < 5 mil	12.83	34
	≥ 5 mil	12.83	34
Gender	Male	57.36	152
	Female	42.64	113
Location	Seoul	57.74	153
	Gyeonggi	16.98	45
	Others	25.28	69
Type of participant	Patient	67.92	180
	Patient guardian	16.60	44
	Nurse	6.79	18
	Doctor	3.77	10
	Others	4.92	13
Perceived socio-economic status	Lower class	6.79	18
	Lower-middle class	15.09	40
	Middle class	54.34	144
	Middle-upper class	18.87	50
	Upper class	4.91	13
Total		100	265

^a^The currency is in Korean won; 1 mil = 1,000,000 Korean won

### Perception of the current ECG testing system

Table 2 presents the results of the descriptive analysis regarding the perception of the current ECG testing system by type of participant. Patients and patient guardians were grouped, and other occupations, such as administrators, were grouped into “others.”

**Table 2:** Perception of the current electrocardiography testing system by type of participant

Factor	Category	Status (*n*)				Subtotal (*n*)
		Patient and guardian	Nurse	Doctor	Others	
Usage experience	Yes	161	10	10	7	188
	No	63	8	–	6	77
Degree of knowledge	Very much	18	8	6	–	32
	Much	47	5	1	3	56
	Average	61	2	3	3	69
	Little	76	1	–	6	83
	None	22	2	–	1	25
Perceived need	Very much	44	12	7	1	64
	Much	69	3	2	7	81
	Average	75	2	1	5	83
	Little	18	–	–	–	18
	None	18	–	1	–	19

Overall, the number of participants who had experience with conventional ECG tests was greater in patient and guardian, nurse, and doctor groups. The number was equal in the “others” group.

The patient and guardian, nurse, and doctor groups had more than a little knowledge of ECG testing, whereas the others group was neutral (factor subtotal *n* = 157, 59.25%). The degree of knowledge of the patient and guardian, nurse, doctor, and others groups was 2.83 ± 1.05, 3.89 ± 1.37, 4.3 ± 0.95, and 2.62 ± 0.96, respectively, with all results higher than the median.

In all of the groups, most participants answered that there was at least more than a little need for current ECG testing (factor subtotal *n* = 228, 86.03%). The perceived need for current ECG testing by the patient and guardian, nurse, doctor, and others groups was 3.41 ± 1.14, 3.68 ± 1.09, 4.60 ± 0.70, and 3.69 ± 0.63, respectively, with all results higher than the median.

When asked why they thought that current ECG testing is useful and needed, most of those who gave their opinion in the open-ended questions provided similar answers “Measuring ECG is the most basic process of knowing about heart diseases” (Participant #13), and “Because the doctor told me to do so” (Participant #45).

For those who thought that current ECG testing was not useful, most of the answers were similar to *“*Because I don’t think it’s doing me much good” (Participant #18), or “I do not know about the test, so I cannot see why it is useful” (Participant #20).

Prior to the regression analyses, a correlation analysis (Pearson) was conducted between usage experience and perceived need, and between the degree of knowledge and perceived need. The correlation results were both significant, with correlation values of 0.35 (*p* < 001) and 0.48 (*p* < 001), respectively. In the regression analysis, perceived need was used as the dependent variable. In the case of the independent variables, experience of ECG testing and degree of knowledge regarding current ECG testing were used. The results of the simple linear regression analysis between the perceived need for current ECG testing and relevant independent variables are the following.

In all regression analysis, (simple and multiple) all of the variables were similar to the normal distribution curve. The statistical analysis was conducted using the 95% confidence level. The simple linear regression analysis results showed that the perceived need of those who had experience with ECG testing was higher than the perceived need of those who had no experience (coefficient = 0.89, *p* < 0.001). In addition, it was found that the higher the degree of knowledge, the higher the perceived need (coefficient = 0.47, *p* < 0.001). *R*^2^ values were 0.13 and 0.23, respectively.

In the multiple linear regression analysis between perceived need for current ECG testing and the relevant independent variables, the socio-demographic factors were adjusted. Collinearity testing was conducted and the results showed that there was no correlation between the independent variables that could cause statistical bias (tolerance limit > 0.01 and variance inflation factor <  100).

The multiple regression analysis results showed that the perceived need of those who had experience with ECG testing was higher than the perceived need of those who had not (coefficient = 0.57, *p* < 0.001). Furthermore, it was found that the higher the degree of knowledge, the higher the perceived need (coefficient = 0.32, *p* < 0.001). The *R*^2^ value was 0.35.

### Perception of remote ECG monitoring

The findings of the descriptive analysis of perception of a telemedicine service in a cardiology ward (remote ECG monitoring) by type of participant are summarized in Table 3.

**Table 3:** Perception of a remote electrocardiography monitoring system by type of participant

Factor	Category	Status (*n*)				Subtotal (*n*)
		Patient and guardian	Nurse	Doctor	Others	
Perceived need	Very much	27	3	1	3	34
	Much	119	5	3	10	137
	Average	63	4	6	–	73
	Little	12	6	–	–	18
	None	3	–	–	–	3
Most important feature^a^	Home monitoring	106	6	9	5	126
	Monitoring while out	51	5	–	1	57
	Monitoring all the time	57	1	1	7	66
	Other	8	6	–	–	14
Usage intentions	Yes	168	10	5	11	194
	No	56	8	5	2	71
Desired service provider	Government	62	2	–	4	68
	Hospitals	147	13	10	8	178
	No matter	15	3	–	1	19
Desired main agent^a^	Public institution	181	12	–	10	203
	Private institution	43	6	10	2	61
Development barriers^a^	Low IT	42	8	–	6	56
	Lack of advertisement	62	3	1	–	66
	Lack of knowledge	58	3	3	1	65
	Fear of low personal information security	53	4	5	4	66
	Other	8	–	1	2	11

^a^This category involves missing values. *IT* information technology

All groups replied that remote monitoring is needed (above average, overall *n* = 171, 64.52%). The perceived need of the patients and guardian, nurse, doctor, and others groups was 3.72 ± 0.82, 3.28 ± 1.13, 3.50 ± 0.71, and 4.23 ± 0.44, respectively, with all results higher than the median.

Patient and guardian (*n* = 106, 47.75%), nurse (*n* = 6, 33.33%), and doctor (*n* = 9, 90.0%) groups answered that the most important feature when developing remote ECG monitoring is home monitoring. The others group thought that remote monitoring should be available all of the time (*n* = 7, 53.85%).

In addition, most participants were willing to use a remote ECG monitoring system (patient and guardian *n* = 168, 75.0%; nurse *n* = 10, 55.56%; others *n* = 11, 84.62%; overall *n* = 101, 73.21%). The doctors group was neutral (*n* = 5, 50.0%).

All of the groups desired the hospital to provide a telemedicine service (patient and guardian *n* = 147, 65.63%; nurse *n* = 13, 72.22%; doctor *n* = 10, 100%; others *n* = 8, 61.54%; overall *n* = 178, 67.17%).

The patient and guardian (*n* = 181, 80.80%), nurse (*n* = 12, 66.67%), and others groups (*n* = 10, 83.33%) thought that public institutions should be the main agents of the telemedicine system (overall *n* = 99, 77.34%), whereas the doctor group thought that a private institution should be the main agent (*n* = 10, 100%).

Most critical barriers to telemedicine development were fear of personal information security, lack of knowledge, and lack of advertisement (each approximately 25%). The perceived need for a remote ECG monitoring system by location is presented in Table 4.

**Table 4:** Perceived need for a remote electrocardiography monitoring system by location

Factor	Category	Status*						Subtotal
		Seoul	Gyeonggi	Gangwon	Chungcheong	Jeonla	Gyeongsang	
Perceived need	Very Much	16	10	2	4	2	–	34
	Much	85	14	6	10	12	10	137
	Average	39	17	3	3	4	7	73
	Little	10	4	2	–	1	1	18
	None	3	–	–	–	–	–	3

*Jeju was excluded as there were no participants from this location

Most of the participants in all locations answered that remote ECG monitoring will be needed more than average in the future. Chungcheong showed the highest score in perceived need with a mean and standard deviation of 4.06 ± 0.66, followed by Jeonla (3.79 ± 0.71), Gyeonggi (3.67 ± 0.93), Seoul (3.66 ± 0.83), Gangwon (3.62 ± 0.96), and Gyeongsang (3.50 ± 0.62).

In open-ended questions regarding the intention to use remote ECG monitoring, replies were the following: “Because you can prevent heart-related diseases at an early stage” (Participant #4), “Because it is efficient and convenient so you will not have to come to the hospital in person more often” (Participant #45), or “If the doctor tells me to do so, I will follow the decision” (Participant #90).

Regarding opinions about why participants do not have the intention to use remote ECG monitoring, most of the answers were similar to “I do not trust the current state of medical technology” (Participant #24).

There were some participants who gave additional insight to account for barriers to developing and implementing telemedicine, such as “Lack of discussion between social parties” (Patient #17) or “Opposition from doctor groups” (Participant #21).

## Discussion

### General implications

The group of elderly participants (60 years or older: 26.79%) was the largest of all of the age groups. In addition, 11.72% needed their guardian to fill in the questionnaire on their behalf. This implies that the elderly’s opinion of telemedicine service should take some credit for overall successful implementation of telemedicine. This result is also supported by prior research that has already stated the importance of considering elderly people when developing medical information systems [18].

A considerable number of patients were from other parts of the country (25.28%). These included areas where it takes more than several hours or even air/ship transportation to travel to hospital. Remote monitoring and treatment is a vital solution for people in remote areas. This matches recent domestic research that claims that healthcare accessibility in remote districts or provinces is still low, and that patients choose tertiary care centers in the capital in the first place to obtain better medical service quality than they can receive in local hospitals [19]. This has been also supported in terms of hospital finance that more than 30% of hospital revenue comes from patients from other areas other than Seoul [20]. It can be inferred from these results that healthcare accessibility in Korea is low especially in rural areas, which counters KMA’s argument that healthcare accessibility is high in South Korea so that telemedicine is not needed. A considerable portion of the population comes from very remote places, and remote health service/monitoring will be handy to those in need when implemented.

### Implications regarding conventional ECG tests

The correlation analysis, simple linear regression, and multiple linear regression results all showed that usage experience and degree of knowledge positively affected perceived need. These results match previous research that mentions the importance of knowledge of the hospital information system [21], that experience greatly affects promotion [22], and that many usage experiences are needed prior to implementing a new system.

Such results regarding the conventional ECG system provide some insight into telemedicine. Prior experience of usefulness critically affects one’s perceived need. Otherwise people tend to fear something that they do not know; risks and harm are overestimated. However, these obstacles can be overcome if sufficient knowledge is given to the population, which is why the government is attempting pilot projects regarding telemedicine. In conclusion, the results support the implication that more telemedicine pilot projects are needed so that the population can be exposed to more experience and can gain knowledge of telemedicine in order to perceive the need for it. This is supported by a prior research that also stated the importance of exposure to a new field to develop positive reception [23], although the limitation of this reference is that it was not applied to a field similar to this study.

### Implications regarding telemedicine in a cardiology Ward

In total, 64.52% of all participants thought that telemedicine was needed, and 73.21% of participants were willing to use telemedicine services if provided. Results concur with those of prior research studies in the U.S. in which the majority of patients would use telemedicine services [24, 25]. Against KMA’s opinion that the South Korean population does not desire telemedicine service, there were clear and strong needs for telemedicine in main tertiary hospitals.

Specifically, patients are currently requesting remote diagnosis and checkup services from their homes. Based on these results, future telemedicine systems should consider home-based services accordingly. The results also match the statement in recent research that a “smart home” is one of the preferred services by consumers [26]. However, the limitation was that services other than the ones mentioned within the survey were not taken into account.

The majority of patients preferred hospitals and public institutions to be their telemedicine service provider and main telemedicine agent, respectively. This reflects the current dependency and trust among Korean citizens regarding tertiary care centers which have strong public characteristics, and Korean citizens’ desire for low prices in telemedicine. In contrast, doctors thought that private hospitals should be the main provider and agent. This may have reflected the self-interest of the doctors. That is, most doctors in South Korea eventually become independent and set up their own private medical establishment, and they desire to be key “fee-for-service” providers in the future.

In this research, fear of personal information security (25.0%) ranked top as the main barrier to telemedicine implementation. This finding stands out because based on a prior international report, most patients were comfortable with having all of their health records securely available in the cloud in a telemedicine system [27]. South Koreans seem somehow not that comfortable. This difference may be a result of recent personal information security breaches that have occurred in several major firms and banks recently in Korea. Other barriers such as lack of advertisement (co-top rank, 25.0%), lack of knowledge (24.62%), and low Information Technology (IT) (21.21%) were also important challenges to consider as these barriers were also mentioned in a prior research by Touati [28]. Limitations were that other possible barriers were not taken into consideration. In addition, three critical barriers “lack of advertisement,” “lack of knowledge,” and “fear of personal security” showed similar rates of approximately 25% may implying that the true issue may be “lack of awareness.”

Perceived need was dominant in remote areas farther than the outer regions of the capital. However, another important point was that the need was also high among residents in Seoul, which indicates that high medical accessibility is not related to the lack of need for telemedicine. Remote monitoring systems have the potential to enhance convenience, lower the price, and save time for all users regardless of location. This result is supported by Tozzi [29], who has shown in early research that telemedicine was needed in places even where hospitals were nearby.

## Conclusion

Findings in this study oppose the Korean Medical Association’s opinion that the population is against enforcing telemedicine related laws. Reflection of an up-to-date perception of telemedicine among patients and medical professionals in a tertiary care centers’ cardiology ward was also included in this study. Moreover, the study provides a counter baseline of positive need for telemedicine based on a mixed method of both quantitative and qualitative approaches to overcome past failures caused by the KMA, arguing that the population is against it. This study also provides some details as to what to include in a telemedicine service, which is envisioned to help successfully implement telemedicine in South Korea if it were to come. Future research may include expanding analysis to secondary and primary hospitals for further generalization.

## Additional file

Additional file 1: Semi-Interview Survey Questionnaires. English language copy of the Interview Guide used to direct discussions in this study. (DOCX 17 kb)

## Abbreviations

ECG: Electrocardiography
IRB: Institutional Review Board
IT: Information Technology
KMA: Korean Medical Association
MOU: Memorandum of Understanding
SPSS: Statistical Package for the Social Sciences
