# Supplementary material for: Accepting telemedicine in a circulatory medicine ward in major hospitals in South Korea: patients’ and health professionals’ perception of real-time electrocardiogram monitoring
Source: BMC Health Serv Res. 2018 Apr 20;18:293. doi: 10.1186/s12913-018-3105-y (PMC5910594; doi:10.1186/s12913-018-3105-y)
Supplement: Supplementary file 1 — Semi-Interview Survey Questionnaires. English language copy of the Interview Guide used to direct discussions in this study. (DOCX 17 kb) [file 12913_2018_3105_MOESM1_ESM.docx]

**<Semi-Interview Survey Questionnaires>**

| **Responder Number** |  |  |  |
| --- | --- | --- | --- |

- The contents here will NOT be used other than statistical analysis for researches purposes, under the Korean Statistics Law clause 33 -

| **Greetings,**  Thank you for your precious time that you have provided to answer this survey.  This survey has been developed to research [perception of telemedicine (such as real-time electrocardiogram monitoring) in a circulatory medicine ward in major hospitals in South Korea among patients and health professionals]. The results and visions derived from the analysis of this survey will be used for better telemedicine services for patients in the future. Also, they will be used as baseline data for medical professionals to better provide high quality ECG tele-monitoring services.  Please note that all questionnaires are anonymous, and we **will not use any personal information in any way**.  We plead you to take your time to answer these questions thoroughly  **We wish you ever prosperity and well-being.**  Feb, 2016  **The Catholic University of Seoul** |
| --- |
| You have the right to discuss any matters regarding this research with the Institutional Review Board of the Catholic Medical Center (Main Headquarters).  Under the rights of being a research participant, if you have any unsolved questions, worries, or complaints with the researchers, please contact us using the information provided below.  -**Institutional Review Board of the Catholic Medical Center (Main Headquarters)**: (02) 2258-8196~8205  -**The Catholic University of Seoul, Research Participant Help Desk**: (02) 2258-8196~8205 |
| **Head of Research**: Jae-Hoon, Han / [jh.han531@gmail.com](mailto:jh.han531@gmail.com) / Planning Team, Catholic Medical Center, 222, Banpo-daero, Seocho-gu, Seoul, Republic of Korea |

- Semi-Interview Survey Questionnaires

| 1-3A. Please write a short statement about why you think that conventional ECG testing is not needed. |
| --- |
|  |

| 1-3B. Please write a short statement about why you think that conventional ECG testing is needed. |
| --- |
|  |

(Part of 2.2) Please write other functions of remote monitoring that you think are important:

_________________________________________________________________________________________________________

| 2-3A. Please write a short statement about why you will use remote ECG monitoring. |
| --- |
|  |

| 2-3B. Please write a short statement about why you will not use remote ECG monitoring. |
| --- |
|  |

(Part of 2.6) Please write other causes that you think hinder remote ECG monitoring:

________________________________________________________________________________________________________
